# Considerations for Achieving Cross-Platform Point Cloud Data Fusion across Different Dryland Ecosystem Structural States

**DOI:** 10.3389/fpls.2017.02144

**Published:** 2018-01-10

**Authors:** Tyson L. Swetnam, Jeffrey K. Gillan, Temuulen T. Sankey, Mitchel P. McClaran, Mary H. Nichols, Philip Heilman, Jason McVay

**Affiliations:** ^1^BIO5 Institute, University of Arizona, Tucson, AZ, United States; ^2^School of Natural Resource and Environment, University of Arizona, Tucson, AZ, United States; ^3^Informatics and Computing Program, Northern Arizona University, Flagstaff, AZ, United States; ^4^USDA Agricultural Research Service, Southwest Watershed Research Center, Tucson, AZ, United States

**Keywords:** lidar, terrestrial laser scanning, structure from motion, sUAS

## Abstract

Remotely sensing recent growth, herbivory, or disturbance of herbaceous and woody vegetation in dryland ecosystems requires high spatial resolution and multi-temporal depth. Three dimensional (3D) remote sensing technologies like lidar, and techniques like structure from motion (SfM) photogrammetry, each have strengths and weaknesses at detecting vegetation volume and extent, given the instrument's ground sample distance and ease of acquisition. Yet, a combination of platforms and techniques might provide solutions that overcome the weakness of a single platform. To explore the potential for combining platforms, we compared detection bias amongst two 3D remote sensing techniques (lidar and SfM) using three different platforms [ground-based, small unmanned aerial systems (sUAS), and manned aircraft]. We found aerial lidar to be more accurate for characterizing the bare earth (ground) in dense herbaceous vegetation than either terrestrial lidar or aerial SfM photogrammetry. Conversely, the manned aerial lidar did not detect grass and fine woody vegetation while the terrestrial lidar and high resolution near-distance (ground and sUAS) SfM photogrammetry detected these and were accurate. UAS SfM photogrammetry at lower spatial resolution under-estimated maximum heights in grass and shrubs. UAS and handheld SfM photogrammetry in near-distance high resolution collections had similar accuracy to terrestrial lidar for vegetation, but difficulty at measuring bare earth elevation beneath dense herbaceous cover. Combining point cloud data and derivatives (i.e., meshes and rasters) from two or more platforms allowed for more accurate measurement of herbaceous and woody vegetation (height and canopy cover) than any single technique alone. Availability and costs of manned aircraft lidar collection preclude high frequency repeatability but this is less limiting for terrestrial lidar, sUAS and handheld SfM. The post-processing of SfM photogrammetry data became the limiting factor at larger spatial scale and temporal repetition. Despite the utility of sUAS and handheld SfM for monitoring vegetation phenology and structure, their spatial extents are small relative to manned aircraft.

## Introduction

Measurement and monitoring of ecological processes are limited by what Levin (1992) termed the ‘problem of pattern and scale’ where linking observations across cells, leaves, plants, community, and ecosystem require exponential amounts of information be transferred between fine and broad spatial scale, short and long temporal scale. Conventional ecological research studies require tens to hundreds of small quadrats or plots between 1 and 100 meters square (m^2^) for enough observations to ensure statistically significant *p*-values for testing hypotheses (Huenneke et al., 2001; Kachamba et al., 2017). This change in scale from individual plot to ecosystem inventory is perhaps the most important concept of remote sensing in the life sciences (Woodcock and Strahler, 1987; Turner, 1989; Turner et al., 1989).

Dryland ecosystems, characterized by sparse or patchy vegetation and long periods of senescence, are punctuated by short periods of rapid growth following seasonal precipitation events. Therefore, characterization of both spatial and temporal patterns of vegetation abundance requires both high spatial resolution and temporal repetition over short periods of time. For instance, landscape-scale models of primary production and carbon-uptake should be more accurate when the vegetation abundance is frequently represented at a fine, plant-scale spatial resolution. Recent advances in sensor and platform technology appear to bring these resolutions within reach, but better understanding of detection bias and accuracy are needed, as well as examining the potential for combining multiple sources in a data-fusion approach that builds on strengths of one system to avoid the weakness of the other. For example, measuring the height and volume of an individual tree or shrub requires accurate minimum elevation (bare ground), apical leader, and crown diameter measurements. Given these requirements, the data may become unusable if bare ground is obstructed or incompletely illuminated. In these situations, multiple observations from different platforms may be required to generate a better estimate.

Light detection and ranging (lidar) is the predominant technology for measuring vegetation and earth surface phenomena in three dimensions (3D) (Glennie et al., 2013; Harpold et al., 2015). Manned aircraft equipped with lidar now survey local areas to entire ecosystems or countries (Higgins et al., 2014; Stoker et al., 2016), while terrestrial lidar is used to collect data at sub-centimeter resolution over hectare size areas. Critically, the spatial scale needed for managing at landscape scale can be provided by manned aerial lidar but the scale needed for monitoring ecosystem process often requires resolution that can only be provided by terrestrial lidar.

Advancements in computational processing power and machine vision also allow 3D object reconstruction from nadir and oblique 2D imagery by so-called structure from motion (SfM) photogrammetry and multi-view stereo (MVS) methods (Westoby et al., 2012; Carrivick et al., 2016; Wallace et al., 2016; Mlambo et al., 2017), hereafter referred to simply as “SfM.” Under the right conditions small unmanned aerial systems (sUAS) with SfM can generate sub-centimeter precision point clouds and create digital elevation models (DEM) and digital surface models (DSM) (Dandois and Ellis, 2010; Dandios and Ellis, 2013; Cruzan et al., 2016). Cruzan et al. (2016) give excellent examples of the capability of small and micro UAS in plant ecology.

In the present study, we compare detection biases amongst different 3D remote sensing techniques (lidar and SfM photogrammetry), from three platforms (ground, sUAS, and manned aircraft) in various dryland ecosystem structural states. We combine the new and less expensive technologies, specifically sUAS SfM, with manned aerial lidar to produce more representative measurements of vegetation height and volume. The objectives of the paper are (1) to identify detection biases amongst technologies for four general feature classes: bare earth (both in barren areas and beneath vegetation), herbaceous plants, low woody shrubs, tall woody shrubs and trees, and (2) explore the potential for combining or fusing observations from any two platforms to overcome the detection bias of another. For Objective 1, we use point cloud-to-cloud inter-comparison and raster-based differencing to establish the detection bias. For Objective 2, we use raster-based differencing and also mesh-to-point cloud differencing to establish improved accuracy by combining data sources.

Current trends in research are moving toward collaborative open data and code sharing (Boulton et al., 2011; Kitchin, 2014; Hampton et al., 2015). Version control systems, like GitHub, offer common repositories for software and code development. In this paper, we provide only abbreviated descriptions of the technical details involving the image processing and point cloud alignment workflows in our main text, and instead provide those details in a supplemental public GitHub repository: https://github.com/tyson-swetnam/srer-wgew. We also provide a quick reference table for acronyms and abbreviations used in this text in the supplemental materials (Table S1).

## Methods

### Study areas and species

**Figure 1:**
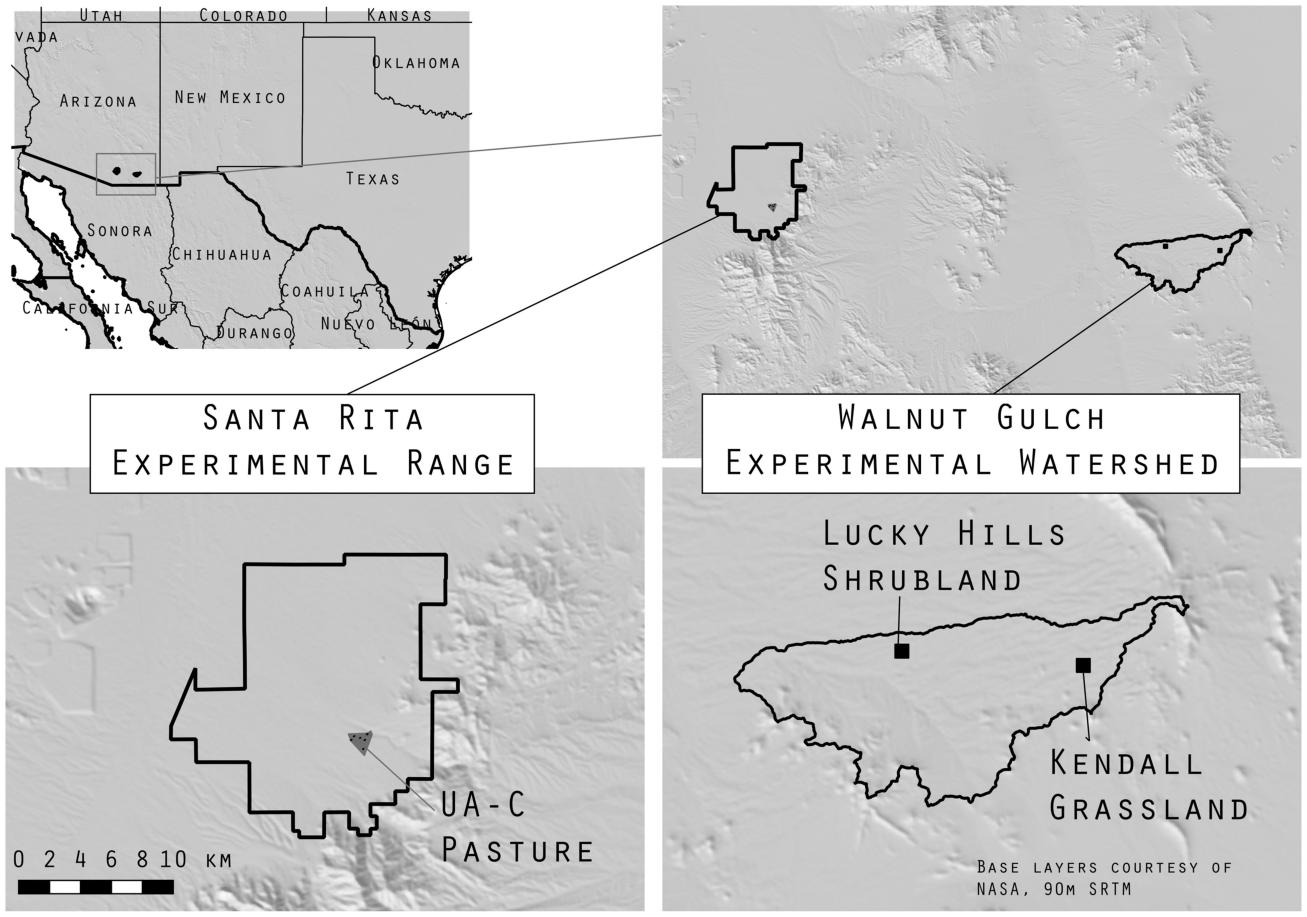
The Santa Rita Experimental Range and Walnut Gulch Experimental Watershed boundaries are shown in black. In the lower left panel the gray triangle is the UA-C pasture, in the lower right panel the two black squares are Lucky Hills Shrubland and Kendall Grassland sites.

The Walnut Gulch Experimental Watershed (WGEW) is a long-term research site surrounding the town of Tombstone, Arizona (30.74° N, −110.05° W) (Figure 1) administered by the United States Department of Agriculture, Agricultural Research Service Southwest Watershed Research Center. The soils in WGEW vary from high in carbonates on the western lower watershed to sandy gravel loams on the eastern upper watershed (Keefer et al., 2008; Osterkamp, 2008; Pelletier et al., 2016). Mean annual temperature in WGEW, measured in Tombstone at 1,384 m a.s.l., is 17.6°C and mean annual precipitation is 300 mm year^−1^. The Santa Rita Experimental Range (SRER) near Tucson, Arizona (31.80° N, −110.84° W) (Figure 1) is administered by the University of Arizona (http://cals.arizona.edu/srer) (McClaran et al., 2003). The SRER soils are characterized as clay loams, sandy loams, and limey upland soils (Brekenfeld and Robinett, 1997). Mean annual temperature and precipitation are 19°C and 358 mm year^−1^, respectively (McClaran and Wei, 2014). Both study sites are typical of dryland ecosystems, characterized as regions where evaporation is 2–5 times greater than precipitation (Safriel et al., 2006).

At WGEW, we worked in two areas: Lucky Hills Shrubland and Kendall Grassland (Figure 1). Lucky Hills is characterized as Chihuahuan desert scrub with shrubs as the dominant life form (Scott et al., 2006; Scott, 2010b). Common species are *Larrea tridentata* (creosote), *Vachellia vernicosa* (whitethorn acacia), *Flourensia cernua* (tarbush), *Parthenium incanum* (mariola), *Rhus microphylla* (little-leaf desert sumac), *Condalia warnockii* (Warnock's Snakewood), *Ephedra triferca* (Mormon tea). The Kendall Grassland site is characterized as semi-arid desert grassland with a mixture of shrub, grass and cactus life forms (Scott et al., 2006; Scott, 2010b). Common species are *Eragrostis lehmanniana* (Lehmann's lovegrass) a non-native grass, and *Prosopis velutina* (velvet mesquite), native grasses include *Hilaria belangeri* (curly mesquite), *Bouteloua eriopoda* (black grama), *B. hirsuta* (hairy grama), and *Aristida hamulosa* (threeawn) (Skirvin et al., 2008), other species include *Yucca baccata* (Banana yucca), *Y. elata* (Soaptree yucca), and *Agave palmeri* (Palmer's agave).

At SRER, we worked in another semi-desert grassland area with mixture of shrubs, grass, and cactus (Pasture UA-C). Common species are *P. velutina, Opuntia engelmannii* (Engelmann's prickly pear cactus), *Acacia greggii* (catclaw acacia), *Mimosa dysocarpa* (velvet pod mimosa), and *Calliandra eriophylla* (false-mesquite), and many species of perennial grass (McClaran et al., 2010; Scott, 2010a).

### Software, code, and computing

Links to the aerial lidar vendor reports, code, and analysis workflows are maintained in a public GitHub repository: https://github.com/tyson-swetnam/srer-wgew. Watershed boundaries and major infrastructure features in WGEW are available from Heilman et al. (2008), Moran et al. (2008), and the SRER data portal: https://cals.arizona.edu/srer/.

For the SfM point cloud creation we used proprietary software packages including: Pix4D (SenseFly, Switzerland) and Photoscan (AgiSoft, 2017). Analysis of point clouds and derivative models were also done in open-source CloudCompare (Girardeau-Montaut, 2011), Point Data Abstraction Library (PDAL, Hobu Inc., 2017) with Docker Community Edition (Merkel, 2014; Boettiger, 2015; Docker Development Team, 2017), and QGIS (Quantum GIS Development Team, 2017). SfM generation of dense point clouds in Agisoft Photoscan took from 1 to 192 h, depending on the number of images used in the reconstruction. Point cloud analyses in CloudCompare were typically less than 5 min per procedure, with large multi-scale model to model cloud comparison (M3C2, Lague et al., 2013) analyses required up to 20 min.

NSF Jetstream cloud computing service (Stewart et al., 2015) was used to filter point clouds for outliers and to translate projections for the aerial lidar and SfM data to common datums using the Docker version of PDAL (docker://pdal/pdal:1.5). PDAL and LAStools (Isenburg, 2014) were also used to remove outliers from the original ^*^.las data files and to compress to ^*^.laz format for online data hosting.

### Instruments and field methods

Characteristics of lidar sensors varied among terrestrial, manned aerial, and sUAS platforms (Table 1). At the Lucky Hills site, the terrestrial lidar survey (TLS) included nine 360 degree scans on October 1, 2015; and at the Kendall grassland site, eight scan and six scan collections were made on September 23, 2015 and October 8, 2015, respectively. Eight terrestrial lidar scans were made at the SRER UA-C site on August 25th, and September 30th, 2016. The individual terrestrial lidar scans from positions located around the AOI insured >50% overlap between scans. Individual scans were mosaicked together during post-processing in Riegl's RiScan Pro software to comprise a total scanned area of ~10 ha with a focus area of interest of ~2 ha (Riegl, 2017). The individual terrestrial lidar scans were aligned using 10 cm diameter reflective cylinder targets mounted on 2 m tall poles. Reflective targets for each scan were centered on rebar driven into the ground and their locations were measured with a survey-grade Trimble GPS system in UTM coordinates tied to existing Coast and Geodetic Survey benchmarks on the watershed (Trimble, 2013). The Trimble R10 (Trimble, 2012) had a measurement error atop ground control points (GCP) of ~5–7 mm in the horizontal and ~6–16 mm in the vertical dimension. At the SRER, stable rebar GCPs were established at 13 locations and 17 cm diameter round plastic lids painted black & white in a cross pattern were temporarily fixed to the rebar rods. We surveyed the GCPs with a Real-Time Kinematic GNSS on October 7, 2016.

**Table 1:** Lidar platforms (TLS-terrestrial lidar scanner, ALS-Aerial lidar scanner, sUAS small Unmanned Airborne System), sensors, collection pulse rate in kilohertz (kHz), laser divergence (mrad), heigh above ground level (AGL), pulse size (centimeters [cm]), root mean square error (RMSE) in the vertical (z) and horizontal (h) plane (cm), laser pulse return density per meter square (ppsm), and the sites of deployments: Santa Rita Experimental Range (SRER) and Walnut Gulch Experimental Watershed (WGEW).

**Platform**	**Sensor**	**Pulse Rate (kHz)**	**mrad (@1/e)**	**AGL (m)**	**Pulse size (cm)**	**RMSE_z_ (cm)**	**RMSE_h_ (cm)**	**ppsm**	**Sites**
TLS	Riegl VZ-400	1200	0.3	2	~ 0.2	±1	±1	1 to 110,000	WGEW, SRER
ALS	Leica ALS-50	48–104	0.15	900	~13.5	±6.4	±50	8 to 25	SRER
ALS	Leica ALS-70	500	0.15	900	~13.5	±8.0	±36	8 to 34	WGEW
sUAS	Velodyne-32	700	3.0 (h), 1.2 (v)	80	~3.0	±89–169	±55	35 to 115	WGEW

Manned aerial lidar scanning (ALS) data was collected by *Woolpert* Inc. over all of WGEW (~50 km^2^) on September 16–18, 2015. The lidar was georeferenced and geometrically corrected using a Real-Time Kinematic GNSS survey as well as a Rapid-Static GPS survey (see Supplemental Materials). Manned aerial lidar were gathered at the SRER in April 2011 by *Sanborn* Inc. The SRER data was georeferenced and geometrically corrected using GNSS Survey (see Supplemental Materials). The positional accuracy of the manned aerial lidar given by the vendors was 6.6–8.0 cm RMSE_z_ for bare earth, and 36–50 cm RMSE_z_ for vegetation (Table 1), meeting the USGS quality level 1 standard (Heidemann, 2012).

An octocopter sUAS (Service-Drone, Germany) equipped with Velodyne-32 lidar sensor was deployed at the WGEW in October, 2016 (Velodyne Acoustics, Inc., Morgan Hill, CA) (Table 1). The octocopter sUAS weighs 5.5 kg and was developed to carry an additional heavy payload of up to 6.5 kg, for a maximum takeoff weight of 12 kg (Sankey et al., 2017a,b). The lidar data were mosaicked together in ENVI v. 5.3 (Exelis Visual Information Solutions, Boulder CO, 2010).

Characteristics of the cameras used for SfM varied (Table 2). Collections from hand held photography focused on either specific individual plants (grasses, cacti, mesquite) or along 30 m transect lines within the SRER UA-C pasture plots. The DJI Osmo was held on a 1 m range pole overhead with the camera pointed approximately nadir and 20–40° oblique. Both still photos and UHD (4 k) video were recorded along transects. Individual frames were extracted from the videos using ffmpeg (FFmpeg Developers, 2016). The rate at which frames were extracted from video varied, given the forward speed of the collection (~1–2 m/s). Typically, 3–6 frames per second were enough to ensure 80% overlap between frames (4 k videos were shot at 24 and 30 frames per second). Quadcopter sUAS were flown over the SRER in summer 2016 using the DJI Phantom 3 and Phantom 4, each weighing ~1.5 kg (Gillan et al., 2017). A third party software Altizure for DJI (Everest Innovation Technology, 2017), was used to program the flight missions with 80% forward and side overlap of the imagery.

**Table 2:** Structure from Motion, platforms, sensors, image size [megapixels (M)], estimated Ground Sample Distance (GSD) pixel size, and SfM photogrammetry points per square meter (ppsm), and the sites of deployments: Santa Rita Experimental Range (SRER) and Walnut Gulch Experimental Watershed (WGEW).

**Platform**	**Sensor**	**Image size**	**GSD (mm)**	**SfM ppsm**	**Site**
Handheld Sony	Sony Exmor a6000	24.3 M	<4	>100,000	SRER
Handheld DJI Osmo	DJI Micro 4/3	16 M	<4	>100,000	SRER
sUAS DJI Phantom 3/4	Sony Exmor	12.4 M	10	5,000–34,000	SRER
sUAS FireFLY6	Sony Exmor a6000	24.3 M	20	1,000–4,000	SRER
sUAS Ebee	SensFly multiSPEC 4C	1.2 M (4 bands)	150	5–140	WGEW

The fixed-wing FireFLY6 flights were performed at the SRER UA-C site on March 17, 2016 and June 28, 2016 at an average flight altitude of 110 m AGL with 65 and 75% forward and side overlap between image tiles. Most of the aerial images were collected facing approximately nadir because the camera is fixed to the belly of the FireFLY6. Pitch, yaw, and roll during the flights resulting in images 0°–15° degrees off nadir.

The eBee is a fixed wing (140 cm wingspan, 750 g mission weight), electric platform with a single pusher propeller at the rear (SenseFly, Switzerland). Flights were performed at an average altitude of 110 m AGL with 80% forward overlap between successive image and 70% side overlap respectively between adjacent flight lines at Kendall grassland site on October 8, 2016. The multispectral Sensfly multiSpec 4C camera records four bands: green (520–580 nm), red (630–690 nm), red edge (720–750 nm), and NIR (760–820 nm). The geo-located, spatially paired, and radiometrically calibrated images were combined to generate an orthomosaic and DSM of the entire flight area in Pix4D (SenseFly, Switzerland). Separately a 3D point cloud was generated for each band and then merged together to generate a single point cloud dataset. The eBee sUAS multispectral images were processed in eMotion and Pix4D (SenseFly, Switzerland) and in Photoscan (AgiSoft, 2017).

#### *Post-hoc* point cloud registration

Comparing two or more point clouds required alignment using at least three GCPs shared between datasets. In the case of our sUAS plots, 10-13 GCPs were needed to ensure high enough accuracy (<5 cm) to successfully detect vegetation change over time (Gillan et al., 2017).

We did not establish GCPs for the eBee SfM photogrammetry in WGEW or the FireFLY6 in SRER. Features that were clearly identifiable in the terrestrial lidar or manned aerial lidar data, e.g., eddy-covariance flux towers, water sampling flumes, fence posts, road features, and small boulders were used *post-hoc* to align point clouds in CloudCompare (Girardeau-Montaut, 2011) with the translation/rotation tool and three-point-picking tool.

### Detection bias

For bare earth analyses locations with barren ground that were clearly observable by the terrestrial lidar were used as references, i.e., roadways and bare patches with no herbaceous component. For the vegetation analyses locations scanned with the terrestrial lidar with no obstruction of the bare earth at the base of the reference plant up to its apical leader were used as references. Terrestrial lidar reference point cloud data were not used in areas with high incidence of occlusion and shadowing due to increasing distance from the scanner.

#### Raster differencing

We produced DEM of Difference (DoD) (Lane et al., 2003; Pelletier and Orem, 2014) for the minimum elevation DEMs and maxima elevation DSMs. DEMs were generated by “cloth simulation filter (CSF)” (Zhang et al., 2016) and “Rasterization” tools in CloudCompare (Girardeau-Montaut, 2011). DEMs were generated using the minimum height function, or bare earth classified points (Class 2, Heidemann, 2012), in the case of the aerial lidar. The DSMs use a maximum height function after outlier points are removed. DEM and DSM rasters were produced at 0.5 m resolution; this was the finest possible resolution for comparison to the aerial manned lidar given its ground return point density. DEM and DSM were exported to QGIS as geotiff (.tif) rasters in their original datum (Table S2). In QGIS we used the raster calculator to generate (a) bare earth DoD_1_: DEM_1_–DEM_2_ (Equation 1); and (b) vegetation DoD_2_: DSM_1_–DSM_2_ (Equation 2) for the different point cloud types. The DoD assess bias within the AOI by establishing the absolute difference between each platform; however DoD differences alone did not intrinsically identify which platform as the source of the bias.

#### Cloud-to-cloud differencing

Lague et al. (2013) created the direct point cloud-to-cloud “multi-scale model to model cloud comparison” (M3C2) tool to compare change across time in point cloud data. The M3C2 is successful at measuring surface and volume differences in geomorphological applications (James et al., 2017; Midgley and Tonkin, 2017). M3C2 computes local distances between “core points” from a “reference” cloud (Lague et al., 2013), we used the terrestrial lidar as the reference cloud and the other clouds as the “test” for differencing. M3C2 uses a “normal” flat surface to estimate the confidence interval based on cloud roughness and registration error (Lague et al., 2013). The difference between two points can be measured in the vertical (z) or horizontal (x and y) axis, or as a linear multi-scale distance based on the normal. In our analysis we compared the vertical (z) change. The significance of the measure is based on the registration error for each core point. Our registration error used the instrument precision of the lidar (Table 1) and the uncertainty of the RTK GNSS (Table S3).

#### Mesh creation and object height measurement

To improve the estimate of vegetation height in a fused data comparison (Objective 2), we used a cloth simulation filter (CSF) technique (Zhang et al., 2016) in CloudCompare (Girardeau-Montaut, 2011), to calculate a minimum elevation (bare earth) model as a mesh. One benefit of the mesh was that it fills space beneath large areas of vegetation where no bare earth surface is remotely sensed. We also used CloudCompare's rasterization tool to generate maximum (DSM) models for object heights. To measure the height of points above the mesh bare earth level we used CloudCompare's Mesh-to-Cloud function.

## Results

### Feature class detection bias

**Figure 2:**
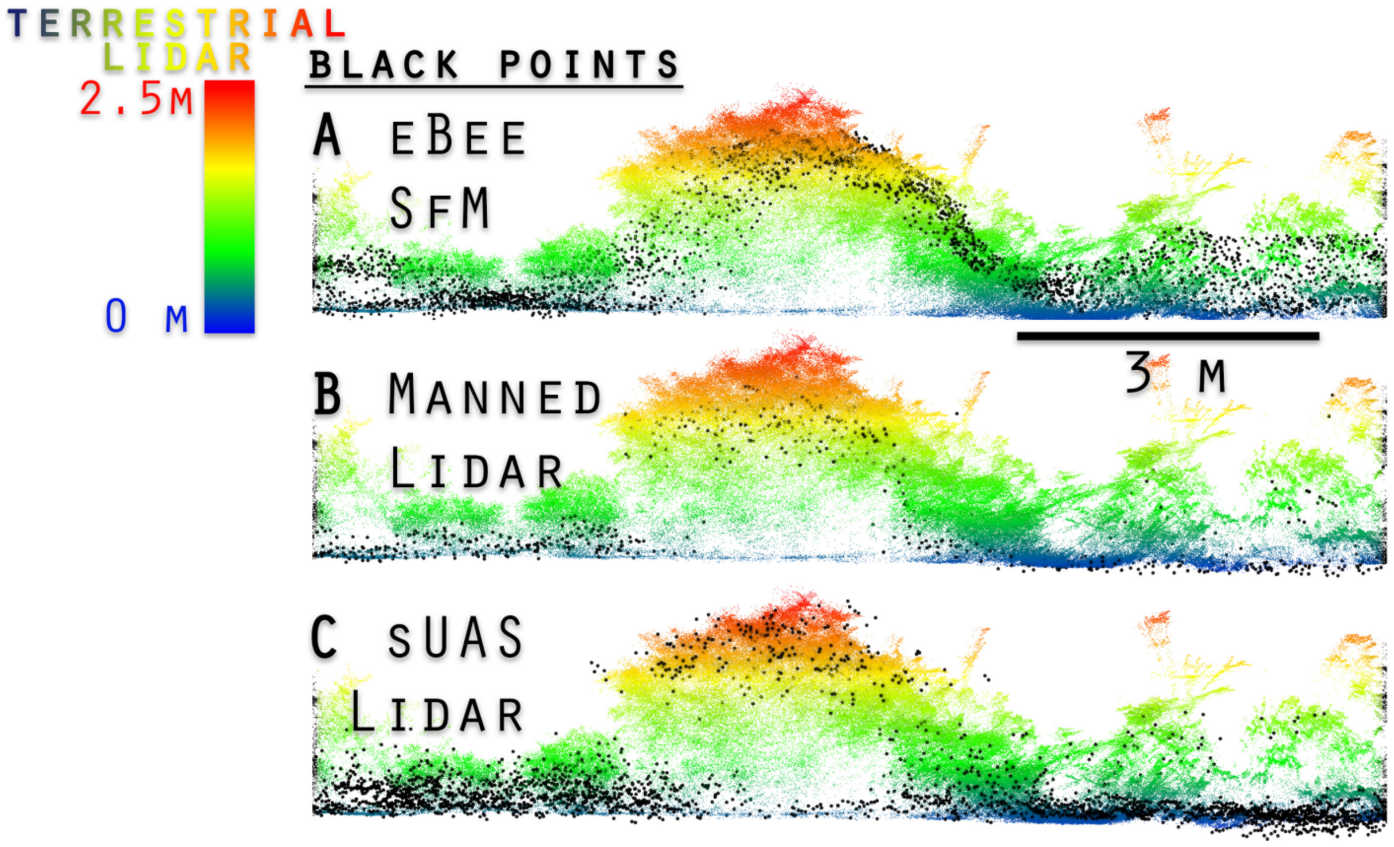
One meter horizontal width transverse view of four point clouds at the WGEW Lucky Hills Shrub site. Terrestrial lidar is compared to eBee SfM **(A)**, terrestrial lidar compared to manned aerial lidar **(B)**, and terrestrial lidar compared to sUAS lidar **(C)**.

Bare earth elevation is only clearly resolved by the terrestrial lidar in barren areas or along roads. The propagated uncertainty (Supplementary Materials) of any observation of bare earth derived from manned aerial lidar to a terrestrial lidar fused measurement was ±8.24 to 9.4 cm RMSE_z_ (Table 3). Aerial lidar (manned and sUAS) was found to be best at detecting bare ground, in part because of its unique point of view, penetrating down through the vegetation. Conversely, the sUAS fails to detect bare ground beneath grass and shrub vegetation (Figure 2), in part due to the GSD of the imagery used to generate it. However, ALS did not always penetrate the canopies of dense leaf-on woody shrubs >2 m tall, nor does it detect sense the fine branches of the shrubs or trees (Table 3). The ALS does resolve larger features on tall woody vegetation (>2 m) with typical underestimates of 5–25 cm (Table 3).

**Table 3:** Vertical (z) differences in point clouds by platform using M3C2 with reference cloud uncertainty of 5 cm; mean ± 99% CI observed difference (cm).

	**Kendall grassland**			**Santa Rita mesquite**		
	**ALS**	**sUAS lidar**	**sUAS SfM^†^**	**ALS**	**sUAS SfM^‡^**	**Hand-held SfM^§^**
Bare ~0 m	2 ± 22	2 ± 73	6 ± 24	5 ± 22	2 ± 16	0 ± 6
Grasses < 1 m	−25 ± 51	0 ± 25	−4 ± 52	n/a	−12 ± 25	−15 ± 15
Shrubs < 2 m	−15 ± 26	−12 ± 25	−16 ± 25	−16 ± 21	−1 ± 32	0 ± 5
Shrubs > 2 m	−31 ± 169	−10 ± 14	−25 ± 13	−18 ± 25	−2 ± 52	−4 ± 13

Negative mean values represent systematic under measurement by the test cloud relative to the terrestrial lidar reference cloud. Positive values are associated with the reference cloud failing to observe elevations at depth, e.g., no returns beneath dense vegetation. n/a value are for no temporal alignment for comparison (Santa Rita). The SfM point clouds derived from:

† *†Sensefly eBee*,

‡ *DJI Phantom 4*,

§ *DJI Osmo*.

The octocopter sUAS lidar had lower positional certainty than the manned aerial lidar, due in part to the sUAS lidar having lower quality GNSS (50+ cm RMSE_z_), and unresolved GPS-IMU drift aboard the octocopter platform (Sankey et al., 2017a,b). When the sUAS lidar point cloud was adjusted using fine alignment tools in CloudCompare its features were comparable to the manned aerial lidar (Figure 2, Table 3). The sUAS lidar resolved both grasses and bare surface in herbaceous and shrubs in the Kendall site, while the manned aerial lidar does not. Despite the increased point density relative to the manned aerial lidar (8–24 ppsm) the sUAS lidar (35–115 ppsm) was still too sparse to identify individual herbaceous plants or shrubs based on only the point cloud.

The Ebee SfM failed at sensing bare earth elevation for areas with dense vegetation on the WGEW. This was not surprising at Kendall Grassland where the herbaceous cover was high. The eBee SfM also tended to under-predict height of woody vegetation in the same way as the manned aerial lidar: it failed to discriminate diffuse outer branches and maximum tree height (Figure 2, Table 3). The initial GNSS referenced eBee SfM point cloud relative to the RTK referenced terrestrial lidar was not accurate and required realignment using the 3-point-picking and fine alignment tools in CloudCompare.

The Phantom 3 and Phantom 4 sUAS SfM photogrammetry also produced point clouds suitable for discriminating large woody vegetation or herbaceous vegetation. However, the resultant SfM point clouds were less accurate than the terrestrial lidar, under-estimating grass height on average by 15 and 4 cm for the larger woody shrubs (Table 3). SfM photogrammetry based on the FireFLY6 imagery was not able to accurately resolve tall woody vegetation (mesquite trees taller than 2 m) on the SRER during the spring leaf-off period, but was improved during the leaf on period in late summer (Supplementary Materials).

### DEM/DSMs of difference for detection bias

Our objective #1 was to establish detection bias between point cloud data and derivatives. In the case of raster data generated from point clouds we establish the difference between the same type of measurement for two different platforms, i.e., DEM_1_-DEM_2_ (DoD_1_) & DSM_1_-DSM_2_ (DoD_2_).

**Figure 3:**
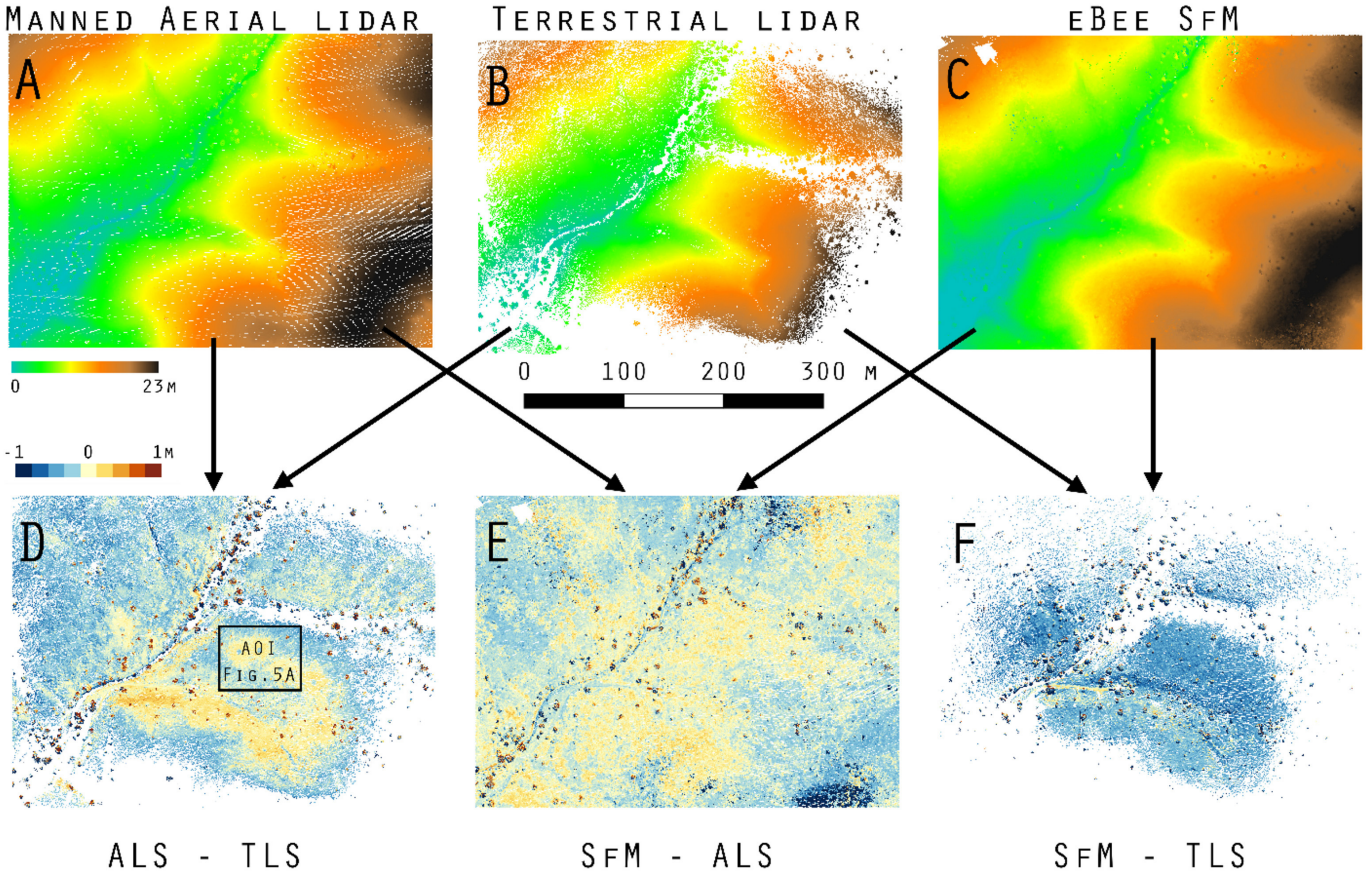
DoD_1_ in minima elevation across Kendall Grassland. **(A)** Manned aerial lidar DEM, **(B)** terrestrial lidar DEM, **(C)** eBee SfM DEM, **(D)** aerial lidar minus terrestrial lidar DoD_1_, **(E)** eBee SfM minus aerial lidar DoD_1_, and **(F)** eBee SfM minus terrestrial lidar DoD_1_.

The DoD_1_ for manned aerial lidar (Figure 3A) vs. terrestrial lidar (Figure 3B) are ±8.24 cm RMSE in areas immediately surrounding the terrestrial laser scan locations in the AOI (Figure 3D). Bare earth elevations are >100 cm different, where bare ground was not visible to the terrestrial lidar sensor (Figure 3D). In these locations the manned aerial lidar and SfM (Figure 3E) are lower (cooler colors) than the terrestrial lidar estimate of bare ground (Figure 3F).

**Figure 4:**
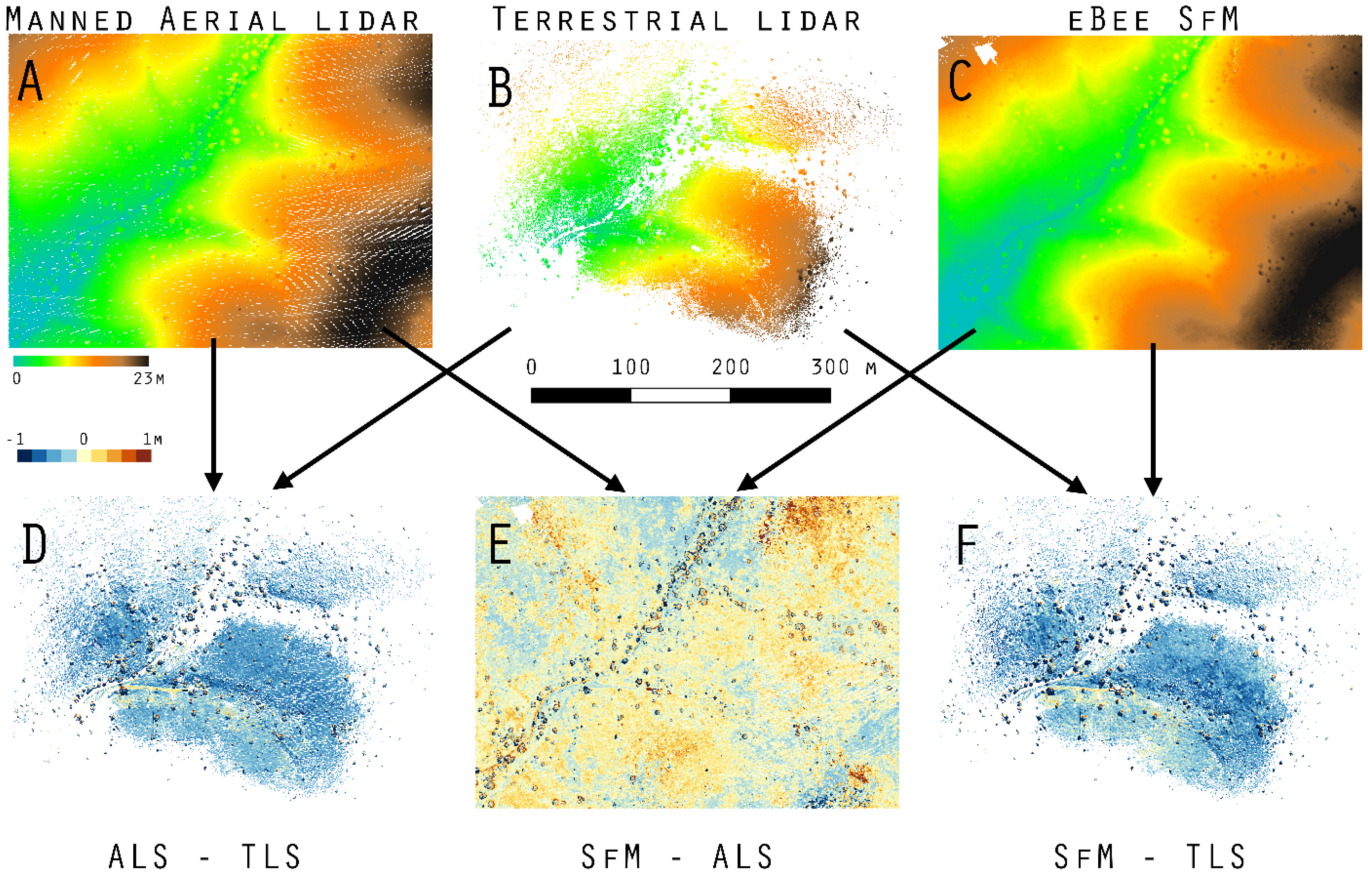
DoD_2_ in maxima elevation across Kendall Grassland. **(A)** Manned aerial lidar DSM, **(B)** terrestrial lidar DSM, **(C)** eBee SfM DSM, **(D)** aerial lidar minus terrestrial lidar DoD_2_, **(E)** eBee SfM minus aerial lidar DoD_2_, and **(F)** eBee SfM minus terrestrial lidar DoD_2_.

The aerial lidar DSM (Figure 4A) under estimate height of grass and woody shrubs by 10 to 120 cm when subtracted from the terrestrial lidar DSM (Figure 4D). There was also a consistent difference across both the SfM-ALS DoD_2_ (Figure 4E), due to the failure of the aerial lidar to sense the herbaceous component. There was an under prediction of height for SfM-TLS DoD_2_ (Figure 4F), with the eBee SfM DSM typically 4 to 52 cm lower than the terrestrial lidar DSM.

### Data fusion accuracy

**Figure 5:**
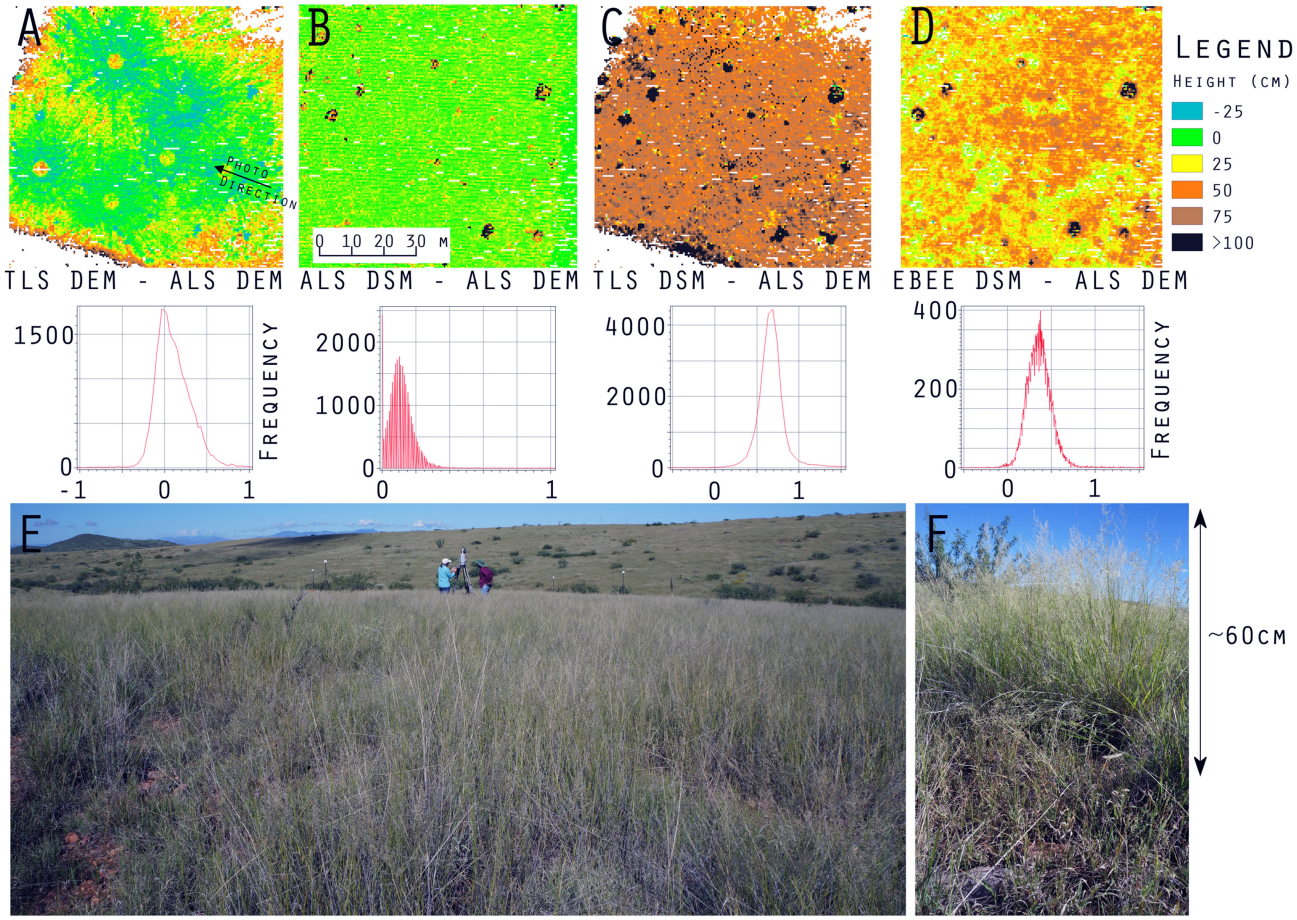
**(A)** DoD_1_ of terrestrial lidar vs. aerial lidar minima also shown in Figure 3, **(B)** DSM difference of aerial lidar minus aerial lidar DEM, **(C)** DSM difference of terrestrial lidar minus aerial lidar DEM, **(D)** DSM difference of eBee SfM minus aerial lidar DEM, **(E)** vegetation on Kendall Grassland 9/23/2015, photograph location, and direction are shown Figure 5A. **(F)** profile of Lehmann's lovegrass on Kendall grassland.

The area immediately around the terrestrial lidar scanner had the most similar bare elevation as aerial lidar DEM (Figure 5A). The associated histogram below Figure 5A shows a mean of approximately zero with standard deviation of 25 cm—similar to the results shown in Table 3 for the M3C2. The weakness of the aerial lidar in measuring vegetation is apparent in Figure 5B where the average height of the point cloud is only 10 cm in grass—an approximate 55 cm under measurement relative to the terrestrial lidar in the same locations (Table 4), visible in Figure 5C. The eBee SfM produced DSM was a more accurate representation of grass height (Figure 5D) when using the aerial lidar derived DEM, and was only 24 cm lower on average than the terrestrial lidar scans (Table 4).

**Table 4:** Average (mean ± 95% CI) object height measurements (cm) taken on the Kendall Grassland site; examples of the aerial lidar (ALS) height model is shown in Figure 5B, the terrestrial lidar DSM minus the aerial lidar DEM (TLS-ALS) in Figure 5C, and eBee DSM minus aerial lidar DEM (SfM-ALS) in Figure 5D.

**Feature classes**	**SfM**	**ALS**	**TLS-ALS**	**SfM-ALS**
Bare ~0 m	5 ± 14	2 ± 16	−1 ± 8	6 ± 8
Grasses < 1 m	20 ± 16	10 ± 12	65 ± 26	41 ± 42
Shrubs < 2 m	114 ± 112	55 ± 124	178 ± 50	87 ± 45
Shrubs > 2 m	27 ± 278	225 ± 24	252 ± 46	177 ± 215

**Figure 6:**
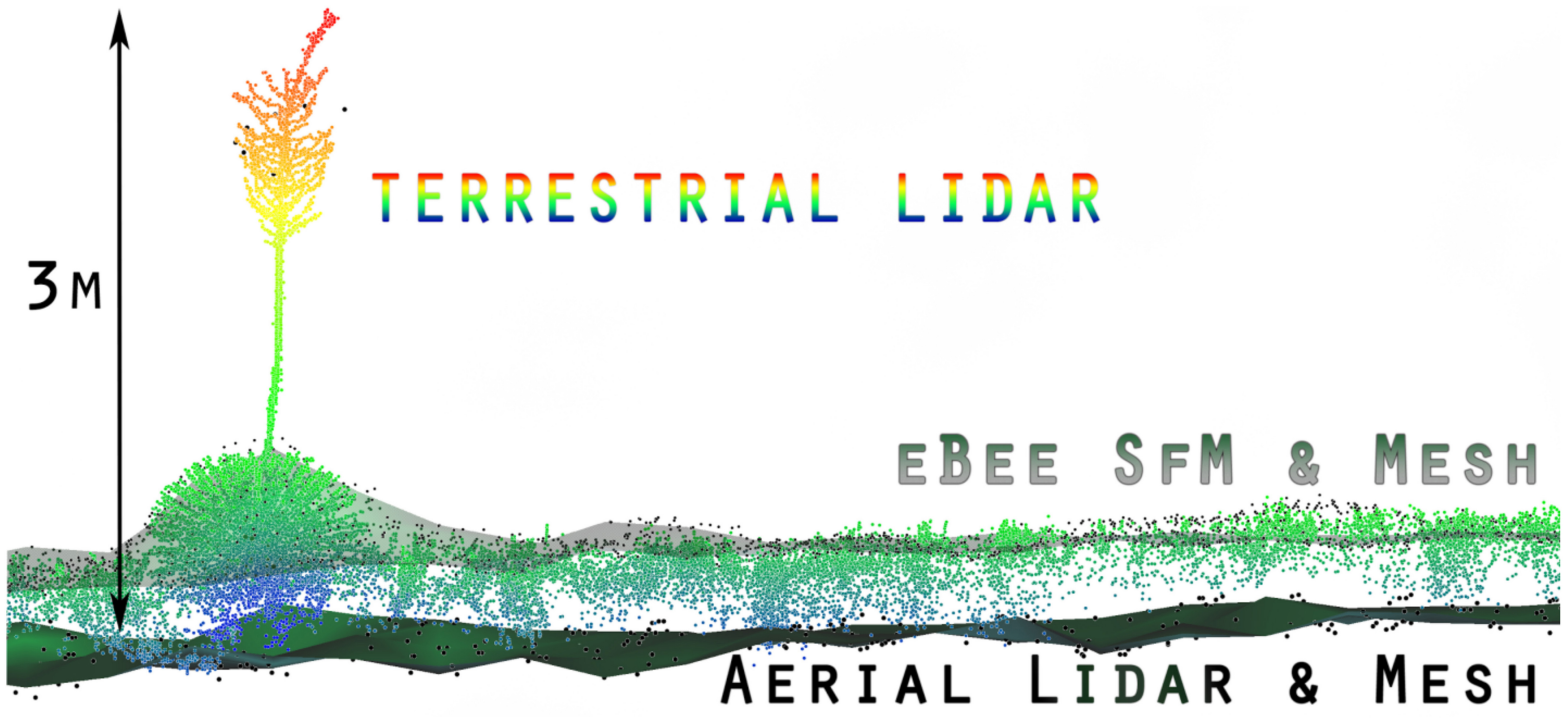
An example of the fusion of three point clouds (two lidar, one SfM photogrammetry) with cloth simulation filter (CSF) mesh (Zhang et al., 2016) to establish bare earth and object height. The terrestrial lidar colored blue to red for height above bare ground, was measured from the lower aerial lidar derived mesh. The upper mesh (transparent green) with the smaller diameter black points are derived from the eBee SfM point cloud. Note, there are no eBee SfM points low in the profile.

Using the mesh-to-cloud height measure in CloudCompare we produced a point cloud based height value for the terrestrial lidar (Figure 6). The bare earth mesh was derived from the CSF (Zhang et al., 2016) (Supplementary Materials). Our anecdotal example, shown in Figure 6, demonstrates one potential method for improving the characterization of vegetation through two platform data fusion: terrestrial lidar and SfM point clouds measure vegetation height above ground but fail to sense bare earth. When they incorporate an aerial lidar point cloud derived bare earth mesh surface the height measure improves. Note in Figure 6 the Ebee SfM points are almost entirely along the top of the grasses and woody shrub (*Yucca elata*, soaptree yucca) as scanned by the terrestrial lidar, while the larger diameter black points from the aerial lidar have few returns in the vertical profile except for the flowering stalk of the yucca. Also note, terrestrial lidar has few or no points low in the vertical profile near ground level. This was due to the flat angle of the horizontal scan profile being obscured by dense herbaceous cover.

## Discussion

No single observation platform solved Levin's “problem of pattern and scale” because each had its own limitations in (1) detecting patterns of important objects in the dryland ecosystem (bare ground, grass, and shrubs), (2) the accuracy of representing the heights of grass and shrubs, and (3) the extent of area represented in a typical data collection campaign. Nonetheless, the combination or fusion of information from the different platforms appears to make greater progress toward solving “problems of pattern and scale” than any single platform alone. Specifically, we suggest the combination of sUAS SfM and either manned or sUAS aerial lidar as providing the best solution at large spatial scales amongst the platforms studied.

To represent spatial and temporal patterns of vegetation, sUAS SfM provides a high quality representation of the presence of grass and shrub vegetation at centimeter scale, but fails to reliably detect bare earth or DEM beneath that vegetation, thus limiting the ability to represent the height and volume of those plants. Aerial lidar on the other hand, provides that needed high quality representation of the pattern of bare earth or DEM. For scale, manned aerial lidar provides spatial resolutions of 0.25–1.0 m^2^, and sUAS Velodyne lidar spatial resolution of 0.1–0.25 m^2^ which is slightly coarser than the terrestrial lidar or sUAS SfM. However, from the perspective of scale of extent or coverage, most aerial lidar data collection campaigns represent hundreds to thousands of km^2^, and there is less need to frequently repeat those campaigns because DEM change in dryland ecosystems are less frequent than the height and volume of grass and shrub vegetation. On the other hand, the extent of a sUAS SfM campaign is at least two orders of magnitude smaller than an aerial lidar campaign, but it is easier and cheaper to repeat several times per year in specific areas of interest, e.g., eddy-covariance flux tower footprints.

### Point cloud bias amongst platforms

The availability of high density lidar point clouds, particularly terrestrial lidar, allowed us to carefully examine differences amongst sUAS lidar, manned aerial lidar, and SfM photogrammetry point clouds. We found the manned aerial lidar, sUAS lidar, and sUAS SfM data all tended to underestimate herbaceous vegetation height in the grassland and shrub areas.

The accuracy of the terrestrial lidar measurements are over an order of magnitude finer than the manned aerial lidar. When comparing the various point clouds to the terrestrial lidar data it was apparent large biases (positional shifts) had been introduced by the various GNSS for both the aerial lidar and SfM photogrammetry. It was also apparent that terrestrial lidar had difficulty resolving bare earth elevations along flat trajectories further away from the scan locations when dense herbaceous vegetation was present (Figures 5, 6). The sUAS lidar was able to penetrate the grass to bare earth elevations and characterize the grass vertical profile, similar to that observed in the terrestrial lidar, but was unable to segment individual plants due to lower density. The manned aerial lidar was not suitable for measuring or monitoring herbaceous grass and small shrubs, as highlighted in Figure 5B. Most of the WGEW manned aerial lidar data are single return at the near ground level in herbaceous grasses (Figure 6). It is likely the ~13.5 cm footprint of the aerial lidar meant smaller features such a grass panicles and small diameter woody stems were not large enough to be detected or were not at the threshold level for discretization from the full waveform data. The largest woody plants, e.g., leaf-on mesquites were detected, but their diffuse branches were not, resulting in an overall 10 to 25 cm height under-estimate of tree height relative to the terrestrial lidar (Figure 5B, Table 4). These results are similar to Luscombe et al. (2014), who compared manned aerial lidar and terrestrial lidar and found similar under-estimates in height for taller vegetation.

In bare ground areas, sUAS SfM data were equivalent to aerial lidar and terrestrial lidar, which is consistent with other recent studies (Lucieer et al., 2012; Westoby et al., 2012; Anderson and Gaston, 2013; Fonstad et al., 2013; James et al., 2017). In dense herbaceous cover SfM photogrammetry point clouds failed to accurately resolve bare ground elevations beneath vegetation accurately. Error in sUAS SfM point clouds are attributed in parts to (1) barrel or pin cushion lens distortion (James and Robson, 2014), and (2) algorithm error when interpreting between bare earth and vegetation (James et al., 2017).

### Influence of canopy cover on DEM generation

We found the sUAS SfM point cloud M3C2 values (Table 3) were all within the uncertainty of the aerial lidar for bare ground (±9.6 cm RMSE_z_) and large woody vegetation (±18 cm RMSE_z_). In the case of the sUAS Phantoms and Osmo SfM, values are within the range of uncertainty of the terrestrial lidar for bare ground (Table S3). Our results were essentially the same as Nouwakpo et al. (2015), who compared soil texture and elevation differences between ground-based SfM and terrestrial lidar and were able to stay within 5 mm RMSE for bare earth patches but not in areas of vegetation with >50% canopy cover.

The sUAS SfM (eBee, Phantoms, Firefly6) data could not be used to generate bare earth elevations beneath herbaceous vegetation (grasses and low shrubs) with any confidence. Another problem in densely vegetated herbaceous sites such as the WGEW Kendall Grassland and the SRER UA-C, occurred when attempting to generate bare earth models with imagery from time periods of vegetation senescence. The resultant models were more likely to be biased because the senescent vegetation cannot be differentiated from soil, such as during the dry season (e.g., the March 2016 Firefly6 flights at UA-C). Standard attempts at removing vegetation using a filter or masking technique, such as NDVI (Cunliffe et al., 2016; James et al., 2017), can only be executed during the leaf-on rainy season in these areas when canopy cover is highest and is spectrally responsive to NDVI.

### Influence of canopy cover on DSM generation

Many recent SfM research papers (Cruzan et al., 2016; Cunliffe et al., 2016; James et al., 2017) report on how SfM does very well in areas with low vegetation cover. In our study, the sUAS SfM was not as accurate at sensing the maximum herbaceous vegetation height as the terrestrial lidar, rather the SfM tended to under predict max heights of the fine herbaceous and woody component. However, SfM at high resolution in hand-held DJI Osmo or low flying Phantoms, was capable of resolving fine herbaceous features and woody stems (Supplementary Materials). Kato et al. (2015) compared SfM vs. terrestrial lidar for tree canopy structure and reported a strong correlation between canopy shape for both technologies. We found similar patterns where the sUAS and handheld SfM reconstruct the shape of large woody canopy shrubs, but are unable to penetrate deeply into the canopy of individual trees to reveal their internal structure.

### Raster differencing (DoD) vs. cloud-to-cloud comparison (M3C2)

The DoD show vertical change in both the bare elevation and maximum height of vegetation in the two surface models (Figures 4, 5); meanwhile the M3C2 was able to diagnose changes in multiscale and horizontal distance both beneath vegetation and across canopy (Table S3). M3C2 was able to more clearly diagnose errors of horizontal change than the DoD which must be inferred by looking at rasters for distinct vertical changes around the vegetation.

Both methods have utility: DoD rasters are useful in eco-hydrology applications, while the M3C2 maybe more useful for geomorphology (bare elevation change), and monitoring of vegetation dynamics (growth, mortality, abundance and composition). Notably, the computational overhead required for M3C2 is orders of magnitude greater than for raster based modeling but is reduced by the use of GPU computing.

### Data management

Our large data sets created a computing challenge when comparing the largest point clouds (>50 GB) in a single computing instance. Cyberinfrastructure storage, such as the CyVerse.org DataCommons (http://datacommons.cyverse.org, Walls, 2017), the NSF lidar repository OpenTopography.org, and USGS National Map are available to users who wish to publically archive processed point clouds. The original images (.JPG or.TIF files) generated by sUAS or handheld collection should be archived at the highest resolution possible, with associated time of flight mission metadata, i.e., GNSS location and camera specifications. One benefit of retaining the imagery for SfM photogrammetry over delivered (i.e., processed discrete) lidar data are their availability for reanalysis with new versions of SfM software which have improved optimization techniques as well as camera and lens correction algorithms. Lidar data, both discrete and full waveform, available in their raw (time-of-flight) format should similarly be stored for future reanalysis.

To help with processing we used CyVerse Atmosphere and NSF Jetstream (Stewart et al., 2015) virtual machine instances. We found the largest instances were ideal for large distributed jobs running point cloud analyses (without GPU). For users who are not able to purchase workstation class PCs, access to cloud or HPC solutions are ideal alternatives at a fraction of the initial start-up cost. We expect the availability of cloud computing and cyberinfrastructure to become more common as computing technology further develops. At the same time, we expect the volume of high-resolution 3D data to increase, as sUAS technology becomes more widely used by geoscientists and ecologists.

Fusion of point cloud data from different sensors and formats presents a problem for ensuring data standards and metadata attribution continue through the life cycle of data. Currently, lidar point cloud data are managed under the LAS standard developed from the American Society for Photogrammetry and Remote Sensing (ASPRS). SfM point clouds, stored as LAS/LAZ files, have fundamental differences from lidar LAS/LAZ, including the lack of scan angle, intensity, or return number.

### Costs (time, human, computing)

The SfM point cloud generation became prohibitive with very large image collections (250–5,000 high quality images) requiring from 12 to 192 h to generate the ultra-high resolution point clouds, also maxing out the available RAM on the workstations without chunking the data into tiles and further increasing processing time. In addition, the photogrammetrist typically had to work with individual images to geo-locate ground control points from anywhere between 4 and 16 h for each model. Conversely, the terrestrial lidar required only a few hours of post-processing by the technicians to resolve the ground-control, typically being completed within 24 h of the initial collection. Aerial lidar data require a significant investment, however that step was shouldered by the contracted vendor and was reflected in the cost of the aerial lidar collection.

## Future outlook

The scale of remotely sensed 3D data from hand held platforms, sUAS, and manned aircraft allows us to ask new questions which 25 years ago were unassailable for Levin (1992). However, we must recognize the strengths and weaknesses inherent to the technology and the data when applying them to the measurement of ecological features. We found aerial lidar from both sUAS and manned aircraft to be more accurate at sensing bare ground elevation than terrestrial lidar or sUAS SfM in dense vegetation, but less accurate at measuring fine and woody herbaceous vegetation. By using the aerial lidar to derive an accurate bare earth model and a second technology (terrestrial lidar, sUAS SfM photogrammetry) to measure herbaceous vegetation height and structure, we were able to better characterize vegetation than by any single technology alone. Quantifying propagation of uncertainty when fusing or combining point cloud (Supplementary Materials) or derivative data is likely to be one of the most important steps researchers take when attempting to make ecologically important measurements.

Newer constellations of “dove” satellites which form “flocks” and record the entire global surface at weekly to daily time scale solve a temporal limitation to remote sensing at scale, with nominal spatial resolution (1–3 m) similar to aerial orthophotography (Butler, 2014; McCabe et al., 2016; Zimmerman et al., 2017). In the United States, nationally available lidar data sets (Stoker et al., 2016) provide bare earth models at 1–2 m resolution. Our methods show how these data could be used for more accurately measuring dense vegetation cover, height, and volume from sUAS derived lidar or SfM photogrammetry data and scaling out spatially with satellite products. Despite the utility of sUAS for monitoring vegetation phenology and structure at orders of magnitude higher resolution, the overhead of computational processing of these imagery can became a limiting factor at progressively larger spatial and temporal resolution, suggesting the need for distributed HPC and cloud based solutions like those provided by Stewart et al. (2015).

Keeping pace with innovation and the breadth of remotely sensed information available for conducting ecological research can be challenging for any single field ecologist, particularly for those looking to add new skills or maintain technical proficiency. Establishing where the limitations of these new remote sensing technology are was one goal of this research. We hope to have revealed a few useful, scalable solutions to these problems through data fusion, and provided insight into improved monitoring programs in dryland ecosystems.

## Author contributions

TLS, TTS, PH, MN, and MM conceptualized and designed the research ideas. TLS, TTS, JG, MN, and JM acquired and analyzed the data. TLS wrote the draft with contributions from TTS, PH, MN, MM, and JG.

### Conflict of interest statement

The authors declare that the research was conducted in the absence of any commercial or financial relationships that could be construed as a potential conflict of interest.

## References

[B1] AgiSoft (2017). AgiSoft PhotoScan. Professional Edn. AgiSoft LLC, St. Petersburg, Russia.

[B2] Anderson K. Gaston K. J. (2013). Lightweight unmanned aerial vehicles will revolutionize spatial ecology. Front. Ecol. Environ. 11, 138–146. 10.1890/120150

[B3] Brekenfeld D. J. Robinett D. (1997). Soil and Range Resource Inventory of the Santa Rita Experimental Range. Tucson, AZ: Natural Resources Conservation Service Available online at: https://cals.arizona.edu/srer/maps/BR1997.pdf

[B4] Boettiger C. (2015). An introduction to docker for reproducible research. ACM SIGOPS Operat. Syst. Rev. 49, 71–79. 10.1145/2723872.2723882

[B5] Boulton G. Rawlins M. Vallance P. Walport M. (2011). Science as a public enterprise: the case for open data. Lancet 377, 1633–1635. 10.1016/S0140-6736(11)60647-8 21571134

[B6] Butler D. (2014). Many eyes on earth. Nature 505, 143–144. 10.1038/505143a 24402262

[B7] Carrivick J. L. Smith M. W. Quincey D. J. (2016). Structure from Motion in the Geosciences. Hoboken, NJ: John Wiley & Sons.

[B8] Cruzan M. B. Weinstein B. G. Grasty M. R. Kohrn B. F. Hendrickson E. C. Arredondo T. M.. (2016). Small unmanned aerial vehicles (micro-UAVs, Drones) in plant ecology. Appl. Plant Sci. 4:1600041. 10.3732/apps.1600041 27672518 PMC5033362

[B9] Cunliffe A. M. Brazier R. E. Anderson K. (2016). Ultra-Fine grain landscape-scale quantification of dryland vegetation structure with drone-acquired structure-from-motion photogrammetry. Remote Sens. Environ. 183, 129–143. 10.1016/j.rse.2016.05.019

[B10] Dandois J. P. Ellis E. C. (2010). Remote sensing of vegetation structure using computer vision. Remote Sens. 2, 1157–1176. 10.3390/rs2041157

[B11] Dandios J. P. Ellis E. C. (2013). High spatial resolution three-dimensional mapping of vegetation spectral dynamics using computer vision. Remote Sens. Environ. 136, 259–276. 10.1016/j.rse.2013.04.005

[B12] Docker Development Team (2017). Version 17.03. Available online at: https://www.docker.com/

[B13] Everest Innovation Technology (2017). Altizure for DJI App. Kowloon: Everest Innovation Technology Available online at: https://next.altizure.com/

[B14] Exelis Visual Information Solutions (2010). ENVI v. 5.3. Boulder, CO.

[B15] FFmpeg Developers (2016). ffmpeg Tool (Version be1d324) [Software]. Available online at: http://ffmpeg.org/

[B16] Fonstad M. A. Dietrich J. T. Courville B. C. Jensen J. L. Carbonneau P. E. (2013). Topographic structure from motion: a new development in photogrammetric measurement. Earth Surf. Processes Landforms 38, 421–430. 10.1002/esp.3366

[B17] Gillan J. K. McClaran M. P. Swetnam T. L. Heilman P. Turner R. (2017). Estimating forage utilization with unmanned aerial imagery, in Poster Presented at Research Insights in Semi-arid Ecosystems (RISE) (Tucson, AZ). Available online at: http://www.tucson.ars.ag.gov/rise/Posters/GillanPoster.pdf

[B18] Girardeau-Montaut D. (2011). CloudCompare-Open Source Project. Grenoble: OpenSource Project Available online at: http://www.cloudcompare.org/

[B19] Glennie C. L. Carter W. E. Shrestha R. L. Dietrich W. E. (2013). Geodetic imaging with airborne LiDAR: the earth's surface revealed. Rep. Prog. Phys. 76:086801. 10.1088/0034-4885/76/8/086801 23828665

[B20] Hampton S. E. Anderson S. S. Bagby S. C. Gries C. Han X. Hart E. M. (2015). The tao of open science for ecology. Ecosphere 6, 1–13. 10.1890/ES14-00402.1

[B21] Harpold A. A. Marshall J. A. Lyon S. W. Barnhart T. B. Fisher B. A. Donovan M. (2015). Laser vision: lidar as a transformative tool to advance critical zone science. Hydrol. Earth Syst. Sci. Discuss. 12, 1017–1058. 10.5194/hessd-12-1017-2015

[B22] Heidemann H. K. (2012). Lidar Base Specification. Techniques and Methods.

[B23] Heilman P. Nichols M. H. Goodrich D. C. Miller S. N. Guertin D. P. (2008). Geographic information systems database, Walnut Gulch Experimental Watershed, Arizona, United States. Water Resour. Res. 44:W05S11. 10.1029/2006WR005777

[B24] Higgins M. A. Asner G. P. Martin R. E. Knapp D. E. Anderson C. Kennedy-Bowdoin T. (2014). Linking Imaging Spectroscopy and LiDAR with Floristic Composition and Forest Structure in Panama. Remote Sens. Environ. 154, (Suppl. C), 358–367. 10.1016/j.rse.2013.09.032

[B25] Hobu Inc. (2017). PDAL: Point cloud Data Abstraction Library. Release 1.5. Copywrite (c) 2017. Available online at: http://www.pdal.io

[B26] Huenneke L. F. Clason D. Muldavin E. (2001). Spatial heterogeneity in chihuahuan desert vegetation: implications for sampling methods in semi-arid ecosystems. J. Arid Environ. 47, 257–270. 10.1006/jare.2000.0678

[B27] Isenburg M. (2014). LAStools - Efficient LiDAR Processing Software (version 141017, unlicensed). Available online at: http://rapidlasso.com/LAStools

[B28] James M. R. Robson S. (2014). Mitigating systematic error in topographic models derived from UAV and ground-based image networks. Earth Surf. Processes Landforms 39, 1413–1420. 10.1002/esp.3609

[B29] James M. R. Robson S. Smith M. W. (2017). 3-D uncertainty-based topographic change detection with structure-from-motion photogrammetry: precision maps for ground control and directly georeferenced surveys. Earth Surf. Processes Landforms 42, 1769–1788. 10.1002/esp.4125

[B30] Kachamba D. J. Ørka H. O. Næsset E. Eid T. Gobakken T. (2017). Influence of plot size on efficiency of biomass estimates in inventories of dry tropical forests assisted by photogrammetric data from an unmanned aircraft system. Remote Sens. 9:610 10.3390/rs9060610

[B31] Kato A. Obanawa H. Hayakawa Y. Watanabe M. Yamaguchi Y. Enoki T. (2015). Fusion between UAV-SFM and terrestrial laser scanner for field validation of satellite remote sensing, in 2015 IEEE International Geoscience and Remote Sensing Symposium (IGARSS) (Milan).

[B32] Keefer T. O. Moran M. S. Paige G. B. (2008). Long-term meteorological and soil hydrology database, Walnut Gulch Experimental Watershed, Arizona, United States. Water Resour. Res. 44:W05S07. 10.1029/2006WR005702

[B33] Kitchin R. (2014). The Data Revolution: Big Data, Open Data, Data Infrastructures and their Consequences. London: SAGE Publishing.

[B34] Lague D. Brodu N. Leroux J. (2013). Accurate 3D comparison of complex topography with terrestrial laser scanner: application to the rangitikei canyon (N-Z). ISPRS J. Photogramm. Remote Sens. 82, 10–26. 10.1016/j.isprsjprs.2013.04.009

[B35] Lane S. N. Westaway R. M. Murray Hicks D. (2003). Estimation of erosion and deposition volumes in a large, gravel-bed, braided river using synoptic remote sensing. Earth Surf. Processes Landforms 28, 249–271. 10.1002/esp.483

[B36] Levin S. A. (1992). The problem of pattern and scale in ecology: the robert H. MacArthur Award Lecture. Ecology 73, 1943–1967. 10.2307/1941447

[B37] Lucieer A. Robinson S. Turner D. Harwin S. Kelcey J. (2012). Using a micro-UAV for ultra-high resolution multi-sensor observations of Antarctic moss beds, in ISPRS - International Archives of the Photogrammetry, Remote Sensing and Spatial Information Sciences XXXIX-B1 (Melbourne), 429–433.

[B38] Luscombe D. J. Anderson K. Gatis N. Wetherelt A. Grand-Clement E. Brazier R. E. (2014). What does airborne LiDAR really measure in upland ecosystems? Ecohydrology 8, 584–594. 10.1002/eco.1527

[B39] McCabe M. F. Houborg R. Lucieer A. (2016). High-resolution sensing for precision agriculture: from earth-observing satellites to unmanned aerial vehicles, in Remote Sensing for Agriculture, Ecosystems, and Hydrology XVIII (Edinburgh).

[B40] McClaran M. P. Browning D. M. Huang C. (2010). Temporal dynamics and spatial variability in desert grassland vegetation, in Repeat Photography: Methods and Applications in the Natural Sciences, eds Webb R. H. Boyer D. E. Turner R. M. (Washington, DC: Island Press), 145–166.

[B41] McClaran M. P. Ffolliott P. F. Edminster C. B. (2003). Santa rita experimental range−100 years (1903 to 2003) of accomplishments and contributions, in Conference Proceedings (Tucson, AZ).

[B42] McClaran M. P. Wei H. (2014). Recent drought phase in a 73-year record at two spatial scales: implications for livestock production on rangelands in the Southwestern United States. Agric. For. Meteorol. 197, 40–51. 10.1016/j.agrformet.2014.06.004

[B43] Merkel D. (2014). Docker: lightweight Linux containers for consistent development and deployment. Linux J. 2014:239.

[B44] Midgley N. G. Tonkin T. N. (2017). Reconstruction of former glacier surface topography from archive oblique aerial images. Geomorphology 282, 18–26. 10.1016/j.geomorph.2017.01.008

[B45] Mlambo R. Woodhouse I. H. Gerard F. Anderson K. (2017). Structure from Motion (SfM) photogrammetry with drone data: a low cost method for monitoring greenhouse gas emissions from forests in developing countries. Forests 8:68 10.3390/f8030068

[B46] Moran S. M. Emmerich W. E. Goodrich D. C. Heilman P. Holifield Collins C. D. Keefer T. O. (2008). Preface to special section on Fifty Years of Research and Data Collection: U.S. Department of Agriculture Walnut Gulch Experimental Watershed. Water Resour. Res. 44:W05SW01. 10.1029/2007WR006083

[B47] Nouwakpo S. K. Weltz M. A. McGwire K. (2015). Assessing the performance of structure-from-motion photogrammetry and terrestrial lidar for reconstructing soil surface microtopography of naturally vegetated plots. Earth Surf. Processes Landforms 41, 308–322. 10.1002/esp.3787

[B48] Osterkamp W. R. (2008). Geology, soils, and geomorphology of the walnut gulch experimental watershed, tombstone, Arizona. J. Arizona Nevada Acad. Sci. 40, 136–154. 10.2181/1533-6085-40.2.136

[B49] Pelletier J. D. Nichols M. H. Nearing M. A. (2016). The influence of holocene vegetation changes on topography and erosion rates: a case study at walnut gulch experimental watershed, Arizona. Earth Surf. Dyn. Discuss. 4, 471–488. 10.5194/esurf-4-471-2016

[B50] Pelletier J. D. Orem C. A. (2014). How do sediment yields from post-wildfire debris-laden flows depend on terrain slope, soil burn severity class, and drainage basin area? Insights from airborne-LiDAR change detection. Earth Surf. Processes Landforms 39, 1822–1832. 10.1002/esp.3570

[B51] Quantum GIS Development Team (2017). Quantum GIS Geographic Information System. Open Source Geospatial Foundation Project. Available online at: https://www.qgis.org

[B52] Riegl (2017). Reigl VZ-400 Data Sheet. Available online at: http://www.riegl.com/uploads/tx_pxpriegldownloads/10_DataSheet_VZ-400_2017-06-14.pdf

[B53] Safriel U. Adeel Z. Niemeijer D. Puigdefabregas J. White R. Lal R. (2006). Dryland systems, in Ecosystems and Human Well-being. Current State and Trends, Vol. 1, (Washington, DC: Island Press), 625–656.

[B54] Sankey T. Donager J. McVay J. Sankey J. B. (2017a). UAV Lidar and hyperspectral fusion for forest monitoring in the southwestern USA. Remote Sens. Environ. 195, 30–43. 10.1016/j.rse.2017.04.007

[B55] Sankey T. T. McVay J. Swetnam T. L. McClaran M. P. Heilman P. Nichols M. (2017b). UAV hyperspectral and lidar data and their fusion for arid and semi-arid land vegetation monitoring. Remote Sens. Ecol. Conserv. [Epub ahead of print]. 1–14. 10.1002/rse2.44

[B56] Scott R. (2010a). AmeriFlux radiological and meteorological data for santa rita mesquite savanna site, Carbon Dioxide Information Analysis Center (CDIAC) Datasets (Tucson, AZ).

[B57] Scott R. L. (2010b). Using watershed water balance to evaluate the accuracy of eddy covariance evaporation measurements for three semiarid ecosystems. Agric. For. Meteorol. 150, 219–225. 10.1016/j.agrformet.2009.11.002

[B58] Scott R. L. Huxman T. E. Cable W. L. Emmerich W. E. (2006). Partitioning of evapotranspiration and its relation to carbon dioxide exchange in a chihuahuan desert shrubland. Hydrol. Processes 20, 3227–3243. 10.1002/hyp.6329

[B59] Skirvin S. Kidwell M. Biedenbender S. Henley J. P. King D. Collins C. H. (2008). Vegetation data, Walnut Gulch Experimental Watershed, Arizona, United States. Water Resour. Res. 44:W05S08. 10.1029/2006WR005724

[B60] Stewart C. A. Cockerill T. M. Foster I. Hancock D. Merchant N. Skidmore E. (2015). Jetstream: a self-provisioned, scalable science and engineering cloud environment, in Proceedings of the 2015 XSEDE Conference: Scientific Advancements Enabled by Enhanced Cyberinfrastructure (St. Louis, MI: ACM), 1–8. 10.1145/2792745.2792774

[B61] Stoker J. M. Brock J. C. Soulard C. E. Ries K. G. Sugarbaker L. J. Newton W. E. (2016). USGS Lidar Science Strategy—Mapping the Technology to the Science: U.S. Geological Survey Open-File Report 2015–1209, 33.

[B62] Trimble (2012) *Trimble R10 GNSS Dat Sheet. Trimble Navigation Limited 10355 Westmoor Dr Westminster CO 80021 USA* Available online at: https://solutions.seilerinst.com/Portals/1/Trimble/TrimbleR10_DS_1012_DataSheetLR.pdf

[B63] Trimble (2013) *Trimble S6 Robotic Total Station Data Sheet. Trimble Navigation Limited 10355 Westmoor Dr Westminster CO 80021 USA* Available online at: http://trl.trimble.com/docushare/dsweb/Get/Document-208580/022543-098L_TrimbleS6_DS_0613_LR.pdf

[B64] Turner M. G. (1989). Landscape Ecology: the effect of pattern on process. Annu. Rev. Ecol. Syst. 20, 171–197. 10.1146/annurev.es.20.110189.001131

[B65] Turner M. G. O'Neill R. V. Gardner R. H. Milne B. T. (1989). Effects of changing spatial scale on the analysis of landscape pattern. Landsc. Ecol. 3, 153–162.

[B66] Wallace L. Lucieer A. Malenovský Z. Turner D. Vopěnka P. (2016). Assessment of forest structure using two UAV techniques: a comparison of airborne laser scanning and structure from motion (SfM) point clouds. Forests 7:62 10.3390/f7030062

[B67] Walls R. (2017). CyVerse data commons, in Plant and Animal Genome XXV Conference. Plant and Animal Genome, 2017 (San Diego, CA).

[B68] Westoby M. J. Brasington J. Glasser N. F. Hambrey M. J. Reynolds J. M. (2012). ‘Structure-from-motion’ photogrammetry: a low-cost, effective tool for geoscience applications. Geomorphology 179, 300–314. 10.1016/j.geomorph.2012.08.021

[B69] Woodcock C. E. Strahler A. H. (1987). The factor of scale in remote sensing. Remote Sens. Environ. 21, 311–332. 10.1016/0034-4257(87)90015-0

[B70] Zhang W. Qi J. Wan P. Wang H. Xie D. Wang X. (2016). An easy-to-use airborne LiDAR data filtering method based on cloth simulation. Remote Sens. 8:501 10.3390/rs8060501

[B71] Zimmerman R. Doan D. Leung L. Mason J. Parsons N. Shahid K. (2017). Commissioning the world's largest satellite constellation, in Conference on Small Satellites. Available online at: http://digitalcommons.usu.edu/cgi/viewcontent.cgi?article=3669&context=smallsat

