# Supplementary material for: Considerations for Achieving Cross-Platform Point Cloud Data Fusion across Different Dryland Ecosystem Structural States
Source: Front Plant Sci. 2018 Jan 10;8:2144. doi: 10.3389/fpls.2017.02144 (PMC5770801; doi:10.3389/fpls.2017.02144)
Supplement: Supplementary file 1 [file DataSheet1.docx]

# Supplemental Materials

**Table S1**: Nomenclature used in the text.

| **Unit/Acronym** | **Name** | **Description** |
| --- | --- | --- |
| ALS | Aerial Laser Scanning | lidar collected from a manned aircraft |
| AMERIFLUX |  | Network of eddy-covariance flux towers in the USA |
| AOI | Area of Interest | Plots or other areas where intense observations were made |
| CHM | Canopy Height Model | height of vegetation above ground level, DSM minus DEM |
| cm | centimeter | 1 centimeter, or 10 mm |
| δ | delta | measurable change in quantity |
| DEM | Digital Elevation Model | Bare earth model derived from lidar or SfM |
| dGPS | differential Global Positioning System | satellite network which triangulates position on Earth surface which also uses a second base station for error correction |
| DoD | DEMs of Difference | Change between two DEM models |
| DSM | Digital Surface Model | Maximum surface elevation height – including vegetation |
| Est | estimated | estimated or predicted quantity |
| GCP | Ground Control Point | a target or surveyed point on the surface |
| GIS | Geographic Information Systems | digital cartography or maps produced in a computer |
| GNSS | Global Navigation Satellite System |  |
| GPU | Graphics Processing Unit |  |
| GSD | Ground Sample Distance | The distance between the centroid of two pixels at ground level. |
| HAG | height above ground | point cloud based measure of object height relative to surface level. |
| IMU | Inertial Motion Sensor |  |
| ha | Hectares | 10,000 meters^2^ |
| km | kilometer | 1,000 meters |
| lidar | Light Detection and Range | laser measurements which return x,y,z position in space |
| m | meter | 100 cm. |
| MAE | Mean Absolute Error |  |
| mm | millimeter | 1 millimeter, or 1/10 cm |
| MSE | Mean Square Error |  |
| MVS | Multi View Stereo | a method for generating topography from SfM. |
| nm | nanometer | 10^-9^ meter, or one billionth of a meter |
| Obs | observed | observed quantity |
| ODM | OpenDroneMap | Open Source project for SfM from sUAS |
| phodar | photogrammetic detection and range | similar to SfM, but involving detailed orthophotographic & map projection techniques |
| RMSE | Root Mean Square Error |  |
| RTK | Real Time Kinetic | Survey technique for establishing location with GPS |
| SDE | Standard Deviation of the Error |  |
| SfM | Structure from Motion | stereoscopic reconstruction of three dimensional objects, without inherent scale or georeferencing |
| σ | sigma | 1 standard deviation |
| sUAS | small Unmanned Aerial System | small aircraft equipped with sensors for measurement |
| TLS | Terrestrial Laser Scanning | ground based laser measurements |
| USDA | United States Department of Agriculture |  |
| USGS | United States Geological Survey |  |

**Table S2**: Lidar collection dates, version and projection information, and sensor platform: small Unmanned Aerial Systems (sUAS), terrestrial laser scanning (TLS), aerial laser scanning (ALS).

| **Location** | **Date** | **LAS** | **Projection** | **ESPG** |
| --- | --- | --- | --- | --- |
| sUAS Lucky Hills  WGEW | 10/8/2015 | 1.2 | WGS84 UTM Zone 12N | 26912 |
| sUAS Kendall  WGEW | 10/8/2015 | 1.2 | WGS84 UTM Zone 12N | 26912 |
| TLS Lucky Hills  WGEW | 10/8/2015 | 1.2 | WGS84 UTM Zone 12N | 26912 |
| TLS Kendall  WGEW | 9/24/2015  10/8/2015 | 1.2 | WGS84 UTM Zone 12N | 26912 |
| TLS Santa Rita  WGEW | 8/25/2016  9/30/2016 | 1.2 | WGS84 UTM Zone 12N | 26912 |
| Manned aerial lidar Walnut Gulch | 9/2015 | 1.4 | WGS84 UTM Zone 12N | 26912 |
| Manned aerial lidar  Santa Rita | 4/2011 | 1.2 | NAD83 State Plane HARN Arizona Central, NAVD88 | 2223 |


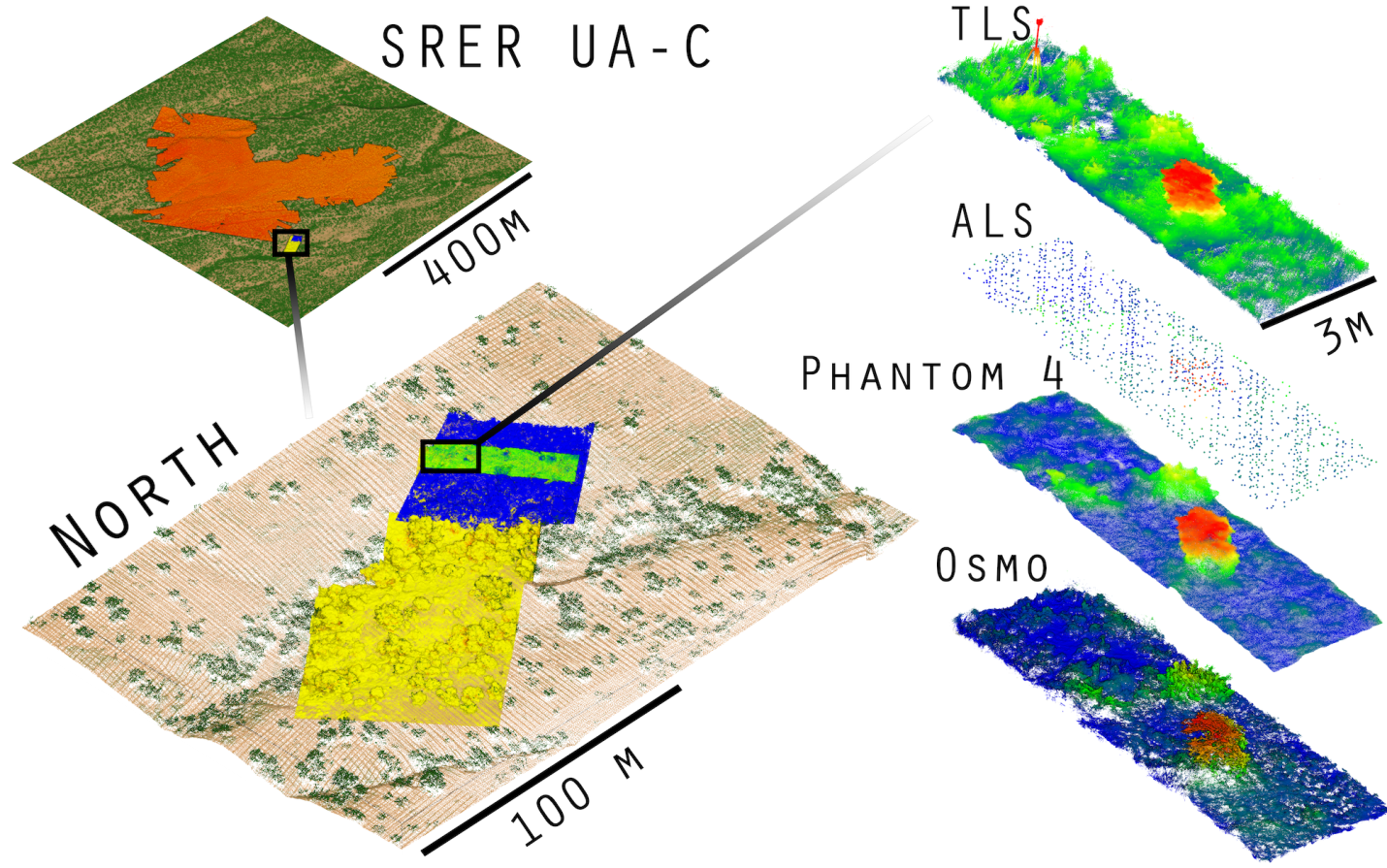


**Figure S1:** Examples of point cloud area and coverage over the SRER UA-C Pasture. Upper left is false color (green to brown intensity from the aerial lidar), the orange cloud is a FireFLY6 SfM point cloud for two flights. In the lower left the same false color aerial lidar is visibly less dense, the yellow is Phantom 4 SfM over ~0.3 ha, the blue area is terrestrial lidar from the Riegl VZ-400, and the green is a single transect from the DJI Osmo hand held SfM. The transects (right side) show point cloud density and height above ground from the top down: terrestrial lidar scan (TLS), aerial lidar scan (ALS), Phantom 4 (P4), and DJI Osmo.

**Statistical analyses**

We took each point observation $\hat{\theta_{i}}$ with an associated error term $e_{i}$ relative to its reference value $\theta_{i}$: $|e_{i}|=|\hat{\theta_{i}}- \theta_{i}|$ [Eq. 3]. The mean square error (MSE) of $\hat{\theta}$ is defined as the expected value of a probability weighted across all samples: $MSE=\sum_{i=1}^{n} {(\hat{\theta_{i}}- \theta_{i})}^{2}$ [Eq. 4], and the related root mean square error (RMSE): $RMSE=\sqrt{\frac{\sum_{i=1}^{n} {(\hat{\theta_{i}}-\theta_{i})}^{2}}{n}}$ [Eq. 5]. We report the standard deviation of the error (SDE): $SDE=\sqrt{\sum_{i=1}^{n} \frac{\left( \bar{(\hat{\theta_{i}}- \theta_{i})}-\left( \hat{\theta_{i}}- \theta_{i} \right) \right)^{2}}{n}}$ [Eq. 6], and mean absolute error (MAE): $\frac{1}{n}\sum_{i=1}^{n} |\hat{\theta_{i}}- \theta_{i}|$ [Eq. 7].

The propagation of uncertainty is calculated as: σ = (δA^2^ + δB^2^+ δC^2^)^1/2^ [Eq. 8], where δA, δB, and δC are the uncertainty from multiple data sources. When two data types are cross-referenced to each other the significance is determined by the “level of detection” or LoD (James et al 2017), such that LoD_95%_ = ± *t ⸱* (δ_TLS_^2^ + δ_X_^2^)^½^ + ε [Eq. 9], where *t* is the confidence interval, e.g. 95% CI *t* = 1.96, and ε is the registration error between the two clouds.

The propagation of uncertainty for the terrestrial lidar following Eq. [8] was 9.85 mm in the horizontal and 19.2 mm in the vertical, which we round to 1 cm RMSE_h_ and 2 cm RMSE_z_, respectively. Positional uncertainty of the ground targets has an additional 2-3 cm of error, for a final estimated δ_TLS_ = 5 cm (SM Table S3).

**Table S3:** Uncertainty estimates for point cloud-to-cloud inter-comparison: LoD_95%_ = ± *t ⸱* (δ_TLS_^2^ + δ_sUAS_^2^)^½^ + ε [Eq. 9], where δ is the instrument uncertainty, *t = 1.96* at the 95% CI, and ε is the registration error, σ is the propagated uncertainty [Eq.8]. For the terrestrial lidar positional uncertainty was estimated from the sensor uncertainty and GNSS RTK error; for the aerial lidar the RMSE was reported by the vendors; and for the sUAS SfM photogrammetry the uncertainty is based on image GSD given in Table 2. Units are in centimeters (cm).

|  | **δ_TLS_** | **δ_sUAS_** | **σ_sUAS_** | **LoD_sUAS_** | **δ_ALSbare_** | **σ_bare_** | **LoD_ALSbare_** | **δ_ALSveg_** | **σ_veg_** | **LoD_ALSveg_** |
| --- | --- | --- | --- | --- | --- | --- | --- | --- | --- | --- |
| **Lucky Hills Shrub** | 5 | 15 | 15.8 | ±31.0 | 8 | 9.4 | ±18.4 | 36 | 36.4 | ±71.2 |
| **Kendall Grassland** | 5 | 15 | 15.8 | ±31.0 | 8 | 9.4 | ±18.4 | 36 | 36.4 | ±71.2 |
| **Santa Rita Mesquite** | 5 | 2 | 5.4 | ±10.6 | 6.4 | 8.2 | ±16 | 50 | 50.3 | ±98.4 |
